# Effects of continuous positive airway pressure therapy on left ventricular performance in patients with severe obstructive sleep apnea

**DOI:** 10.1038/s41598-023-32274-4

**Published:** 2023-04-01

**Authors:** Se-Eun Kim, Jiwon Seo, Younghoon Kwon, Iksung Cho, Chi Young Shim, Jong-Won Ha, Geu-Ru Hong

**Affiliations:** 1 Division of Cardiology, Severance Cardiovascular Hospital, Yonsei University College of Medicine, 50-1 Yonsei-ro, Seodaemun-gu, Seoul, 03722 Republic of Korea; 2 Division of Cardiology, University of Washington, Seattle, WA 98104 USA

**Keywords:** Cardiology, Echocardiography

## Abstract

We investigated myocardial performance concerning obstructive sleep apnea (OSA) severity and the benefits of continuous positive airway pressure (CPAP) therapy. In this randomized sham-controlled trial, 52 patients (mean age, 49 years; 92%, males; mean AHI, 59) with severe OSA were randomly assigned to receive either CPAP or sham treatment for 3 months. The severity of OSA was determined using the apnea/hypopnea index (AHI), oxygen desaturation index (ODI), percentage of sleep time below 90% oxygen saturation (T90), and average O_2_ saturation during sleep (mean SpO_2_). We compared the changes in myocardial work after 3 months of CPAP (n = 26) versus the sham group (n = 26) at rest and during an exercise stress test. Unlike AHI or ODI, indices of hypoxemia including T90 and mean SpO_2_ were significantly correlated with global constructive work, as defined by work of left ventricle (LV) that contributes to LV ejection during systole (T90, β = 0.393, *p* = 0.012; mean SpO_2_, β = 0.331, *p* = 0.048), and global wasted work (GWW), as defined by work of LV that does not contribute to LV ejection (T90, β = 0.363, *p* = 0.015; mean SpO_2_, β =  − 0.370, *p* = 0.019). After 3 months, GWW decreased (80.0 ± 49.2 to 60.8 ± 26.3, *p* = 0.009) and global work efficiency increased (94.0 ± 4.5 to 95.7 ± 2.0, *p* = 0.008) in the CPAP group compared to those in the sham group. At the 3-month follow-up exercise stress echocardiography, worsening of GWW during exercise was significantly decreased in the CPAP group compared to that in the sham group (*p* = 0.045 at 50 W). Hypoxemia indices were closely associated with myocardial performance in patients with severe OSA. CPAP treatment for 3 months improved left ventricular myocardial performance by decreasing wasted work and increasing work efficacy compared to the sham treatment.

## Introduction

Obstructive sleep apnea (OSA) is a common sleep disorder characterized by repetitive upper airway obstruction during sleep^1,2^. Patients with OSA are at an increased risk of cardiovascular diseases^3^. OSA leads to decreased myocardial oxygenation, increased sympathetic activation, and increased afterload, which in turn have adverse effects on both left (LV) and right ventricular (RV) functions^4,5^. Continuous positive airway pressure (CPAP) therapy improves myocardial oxygenation, attenuates sympathetic activation, and reduces the LV afterload^6–8^. CPAP therapy for 3 months improved systolic functions of LV and RV and the  diastolic function of LV^9,10^. LV global longitudinal strain (LVGLS) offers an advantage in evaluating subclinical dysfunction, particularly in heart failure with preserved ejection fraction^11^. However, LVGLS is also affected by the afterload^12^. Thus, decreased LVGLS may be driven by an increased afterload rather than impaired myocardial contractility. While the LV pressure–volume (P–V) loop is the ideal way to evaluate LV function, it is not practical because of its invasive nature^13–15^. Myocardial work is a new echocardiography-based imaging tool that quantifies LV performance based on the pressure–strain (P–S) loop and has been validated by invasively derived P–V measurements in various cardiac conditions. Previous studies reported that the LV P–S loop sufficiently reflects the LV P–V loop, and the LV P–S loop using 2D speckle tracking echocardiography can be an alternative method to evaluate LV function accounting for LV afterload^16,17^. In this study, we tested the hypotheses that the severity of OSA is associated with myocardial performance calculated by myocardial work and that CPAP treatment improves myocardial performance at rest and during exercise.

## Results

### Baseline clinical characteristics

Twenty-six participants from each group were analyzed. Table 1 shows the baseline characteristics of the two groups. There were no significant differences in baseline characteristics between the two groups, except for body mass index, history of dyslipidemia, and proportion of statin users. Baseline PSG characteristics, such as mean apnea/hypopnea index (AHI), mean oxygen saturation (mean SpO_2_) during sleep, percentage of sleep time below 90% oxygen saturation(T90), average heart rate during sleep, and oxygen desaturation index(ODI), which was calculated the average number of desaturation episodes per hour, were not significantly different.

**Table 1:** Baseline characteristics of the study population.

	Sham (n = 26)	CPAP (n = 26)	*p* Value
Age, year	48.8 ± 10.7	49.1 ± 11.4	0.920
Male sex, n (%)	24 (92)	24 (92)	1.000
Height, cm	169.9 ± 7.3	170.9 ± 6.8	0.583
Weight, kg	76.2 ± 12.6	81.7 ± 11.7	0.108
Body mass index, kg/m^2^	26.2 ± 3.0	27.8 ± 2.8	0.047
Body surface area, m^2^	1.90 ± 0.18	1.96 ± 0.18	0.234
Systolic BP mmHg	127.8 ± 16.0	126 ± 19.6	0.722
Diastolic BP mmHg	77.3 ± 10.6	75.8 ± 13.7	0.662
Hypertension, n (%)	15 (58)	17 (65)	0.569
Diabetes mellitus, n (%)	1 (4)	4 (15)	0.158
Dyslipidemia, n (%)	2 (7)	8 (31)	0.035
Current smokers, n (%)	6 (23)	10 (39)	0.229
Medications			
Calcium channel blockers, n (%)	8 (31)	10 (39)	0.560
RAAS blockers, n (%)	8 (31)	9 (35)	0.768
Beta-blockers, n (%)	4 (15)	4 (15)	1.000
Diuretics, n (%)	0 (0)	0 (0)	1.000
Statins, n (%)	2 (7)	8 (31)	0.035
Polysomnographic characteristics			
AHI, events/hour	53.4 ± 20.5	64.2 ± 20.5	0.063
Mean oxygen saturation, %	94.5 ± 1.6	93.9 ± 1.7	0.238
T90, %	7.1 ± 7.3	10.5 ± 11.6	0.210
Average heart rate during sleep, bpm	62.3 ± 6.3	63.3 ± 6.8	0.576
Oxygen desaturation index, events/hour	40.8 ± 20.5	47.7 ± 19.6	0.239

Values are n (%) or mean ± standard deviation.

*CPAP* continuous positive airway pressure, *AHI* apnea/hypopnea index, *BP* blood pressure, *RAAS* renin–angiotensin–aldosterone system, *T90* percentage of sleep time below 90% oxygen saturation.

### Association of severity of OSA with myocardial work

**Figure 1:**
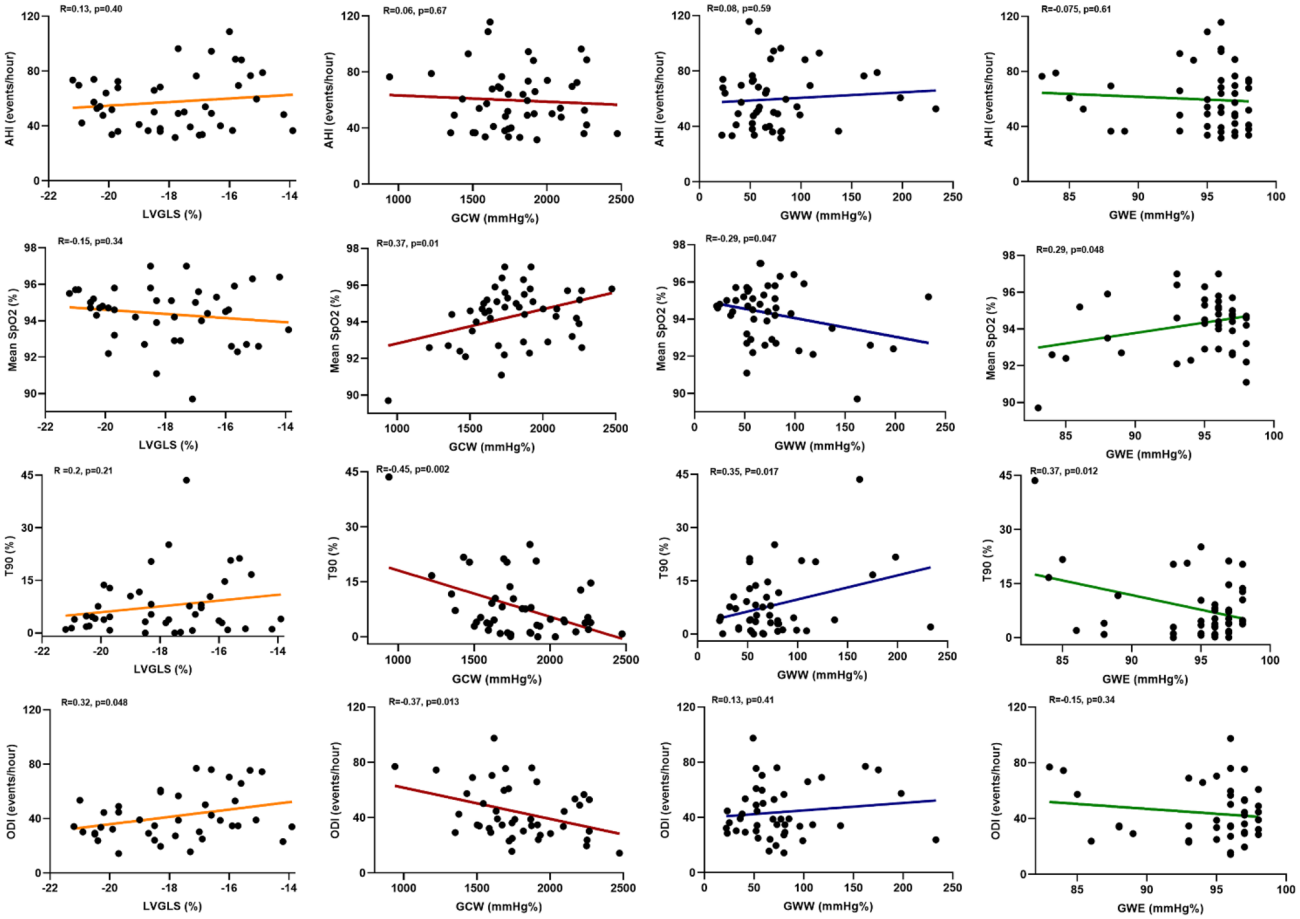
The correlation between AHI and hypoxemia indices (T90, mean SpO_2_, and ODI) with LVGLS and myocardial work. AHI, apnea/hypopnea index; LVGLS, left ventricular global longitudinal strain; GCW, global constructive work; GWE, global work efficiency; and GWW, global wasted work; Mean SpO_2_, mean oxygen saturation; ODI, oxygen desaturation index; T90, percentage of sleep time below 90% oxygen saturation.

Indices of hypoxemia, such as T90 and mean SpO_2,_ were significantly correlated with all myocardial work parameters, except for LVGLS (Fig. 1). T90 was significantly correlated with GWW (r = 0.35, *p* = 0.017), GWE (r =  − 0.37, *p* = 0.012), and GCW (r =  − 0.45, *p* = 0.002). The mean SpO_2_ also correlated with GWW (r =  − 0.29, *p* = 0.047), GWE (r = 0.29, *p* = 0.048), and GCW (r = 0.37, *p* = 0.01). However, AHI was not correlated with either LVGLS or myocardial work parameters, but ODI was correlated with LVGLS (r = 0.32, *p* = 0.048) and GCW (r =  − 0.37, *p* = 0.013). In a multivariate regression analysis including age, sex, body mass index, history of hypertension, and smoking, both T90 and mean SpO_2_ showed significant association with GCW and GWW, but no significant association with AHI or ODI (Table 2).

**Table 2:** Multivariate linear regression analysis* for LVGLS and myocardial work according to AHI and hypoxemia indices (T90, mean SpO_2_, and ODI).

Variables	LVGLS (%)			GCW (mmHg)			GWW (mmHg)			GWE (mmHg)		
	Beta	t	*p* Value	Beta	t	*p* Value	Beta	t	*p* Value	Beta	t	*p* Value
AHI (events/hour)	0.051	0.297	0.768	0.028	0.156	0.877	− 0.053	− 0.313	0.756	0.082	0.508	0.614
T90 (%)	0.113	0.705	0.485	− 0.393	− 2.631	0.012	0.363	2.548	0.015	− 0.352	− 2.559	0.014
Mean SpO_2_ (%)	− 0.128	− 0.757	0.454	0.331	2.040	0.048	− 0.370	− 2.454	0.019	0.282	1.883	0.067
ODI (events/hour)	0.193	1.019	0.316	− 0.323	− 1.08	0.096	0.128	0.688	0.496	− 0.112	− 0.622	0.538

*Adjusted by age, sex, body mass index, history of hypertension, and smoking.

*AHI* apnea/hypopnea index, *LVGLS* left ventricular global longitudinal strain, *GCW* global constructive work, *GWE* global work efficiency and *GWW* global wasted work, *Mean SpO*_*2*_ mean oxygen saturation, *ODI* oxygen desaturation index, *T90* percentage of sleep time below 90% oxygen saturation.

### CPAP effects on myocardial work

Table 3 shows the effects of the sham and CPAP treatment on LV function using echocardiographic parameters. The LV dimension, LV mass index, left atrial volume index, and LV ejection fraction did not significantly different between the baseline and post-treatment. The E’ velocity, an early marker of diastolic dysfunction and LVGLS, was positively changed in the CPAP treatment group after 3 months (change of E’ velocity: 0.65 ± 1.70 cm/s vs. − 0.61 ± 1.85 cm/s, *p* = 0.014). There was no changes in GWI and GCW before or after treatment. However, GWW was decreased (*p* = 0.015) and GWE was improved (*p* = 0.027) after 3 months in the CPAP treatment group but not in the sham treatment group. The GWW and GWE from baseline to the 3-month follow-up significantly differed between the two groups (GWW, *p* = 0.003; GWE, *p* = 0.003) (Supplementary Fig. S1). The changes in GWW and GWE in the subgroups are described in Supplementary Table S1. In patients with hypertension, CPAP treatment decreased GWW and increased GWE. In addition, a subgroup analysis of AHI, mean SpO_2,_ and ODI showed that CPAP treatment decreased GWW and increased GWE significantly in patients with very severe OSA (AHI > 50/h, mean SpO_2_ ≤ 95%, or ODI > 39/h). However, CPAP treatment significantly reduced GWW in the case of T90 ≤ 10% and increased GWE in the case of T90 > 10%, but did not significantly reduce GWW.

**Table 3:** Echocardiographic parameters of the study population.

	Sham (n = 26)		CPAP (n = 26)	
	Baseline	Follow-up	Baseline	Follow-up
Traditional echocardiography parameter				
LVEDV, mL/m^2^	59.3 ± 6.3	57.4 ± 6.2	60.4 ± 9.0	59.5 ± 9.1
LVESV, mL/m^2^	21.3 ± 3.6	20.5 ± 2.7	20.8 ± 4.7	21.4 ± 3.8
LV mass index, g/m^2^	85.7 ± 12.8	83.9 ± 14.1	92.0 ± 15.0	91.2 ± 11.9
LA volume index, mL/m^2^	25.9 ± 5.3	23.8 ± 5.3	26.5 ± 5.8	26.6 ± 5.2
LVEF, %	64 ± 6	64 ± 6	66 ± 5	65 ± 6
S′ velocity, cm/sec	8.4 ± 1.6	9.1 ± 2.0	8.7 ± 1.4	8.9 ± 1.6
e′ velocity, cm/sec	9.4 ± 2.7	8.7 ± 2.9	7.7 ± 2.2*	8.3 ± 2.3
E/e′	7.6 ± 1.5	7.9 ± 1.8	8.9 ± 2.4*	8.9 ± 2.4
2D speckle-tracking indices (LVGLS and Myocardial work)				
LVGLS, %	− 18.0 ± 1.9	− 18.0 ± 2.5	− 17.8 ± 2.1	− 20.0 ± 2.1*^,†^
GWI, mmHg%	1556.8 ± 257.7	1505.0 ± 305.5	1606.8 ± 322.5	1586.1 ± 208.4
GCW, mmHg%	1746.3 ± 258.8	1700.8 ± 354.2	1840.5 ± 347.0	1789.7 ± 212.4
GWW, mmHg%	70.3 ± 39.2	87.4 ± 71.9	80.0 ± 49.2	60.8 ± 26.3^†^
GWE, %	95.3 ± 3.2	94.0 ± 4.3^†^	94.0 ± 4.5	95.7 ± 2.0^†^

*Student’s t-test, *p* < 0.05, compared with the corresponding sham group.

^†^Paired t-test, *p* < 0.05, compared to baseline values within the group.

*GCW* global constructive work, *GWE* global work efficiency, *GWI* global work index, *GWW* global wasted work, *LA* left atrium, *LV* left ventricle, *LVEDV* left ventricular end diastolic volume, *LVEF* left ventricular ejection fraction, *LVESV* left ventricular end-systolic volume, *LVGLS* left ventricular global longitudinal strain.

### CPAP effect on myocardial work during exercise

**Figure 2:**
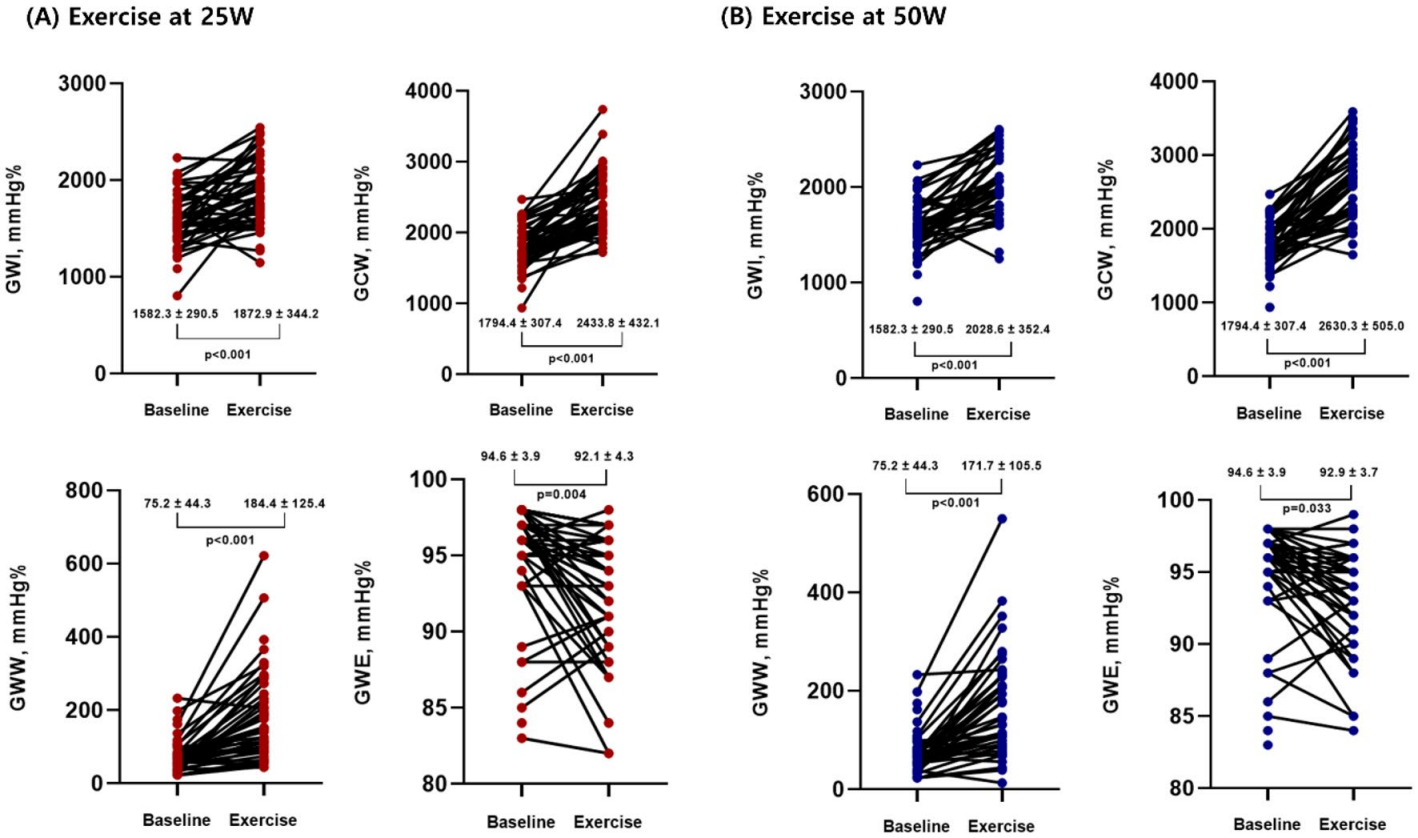
Comparison of myocardial work parameters between baseline and 25 W exercise (**A**) or 50 W exercise (**B**). CPAP, continuous positive airway pressure; GCW, global constructive work; GWE, global work efficiency; GWI, global work index; and GWW, global wasted work.

**Figure 3:**
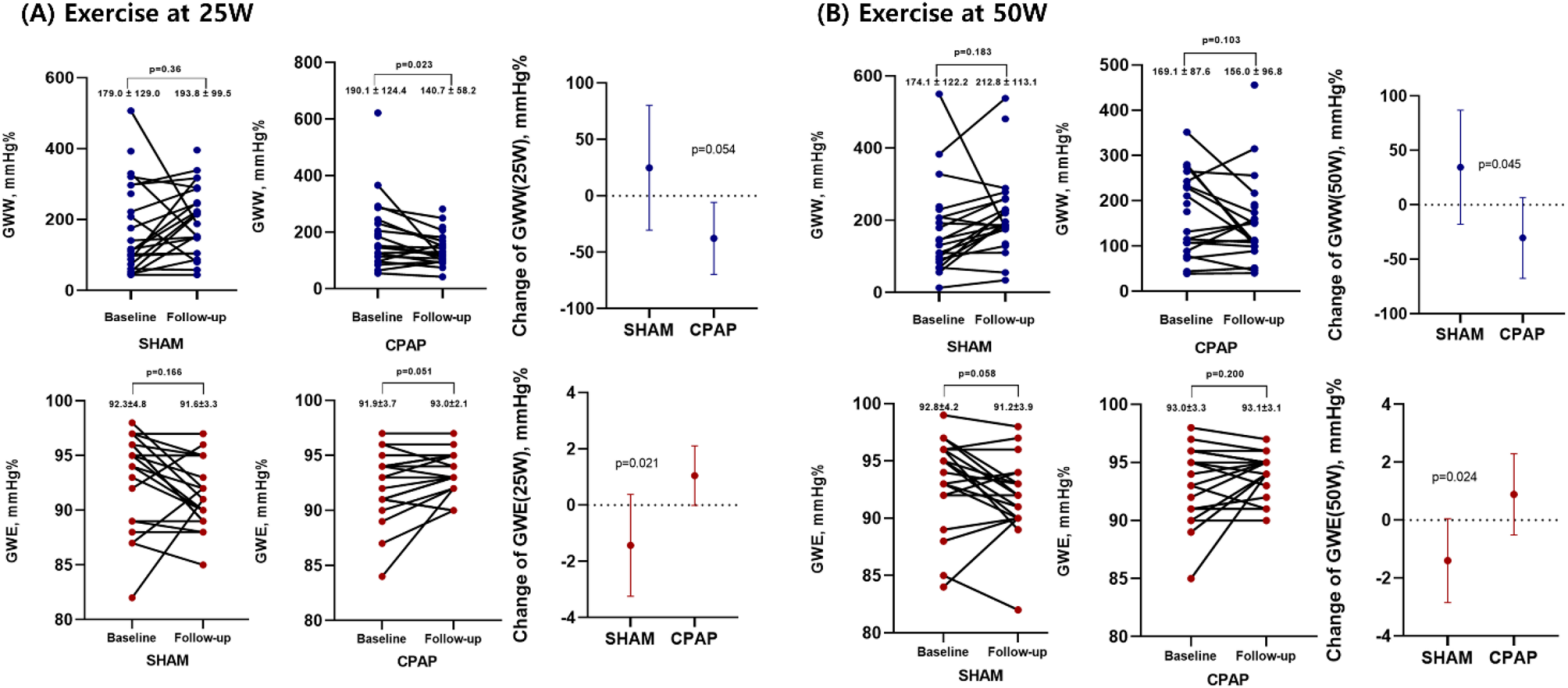
Changes in GWW and GWE in the two study groups according to exercise. (**A**) Changes at 25 W and (**B**) at 50 W. CPAP, continuous positive airway pressure; GWE, global work efficiency; and GWW, global wasted work.

In all patients, GWI, GCW, and GWW were significantly increased during exercise compared to those at baseline for both 25 W and 50 W exercise. However, GWE was decreased during exercise compared to the baseline value (Fig. 2, Supplementary Table S2). During 25 W exercise, there was no difference in myocardial work parameters between the sham and CPAP group before treatment; however, after 3 months of treatment, the GWW of the CPAP group was significantly decreased compared with that at the baseline (190.1 ± 124.4 vs. 140.7 ± 58.2, *p* = 0.023), and it was significantly lower than that of sham group (193.8 ± 99.5 vs. 140.7 ± 58.2, *p* = 0.035). There was no difference in GWE before and after treatment in both groups, but the amount of change was significantly different (*p* = 0.021) (Fig. 3, Supplementary Table S3). After the 50 W exercise, there was no change in pre- and post-treatment myocardial work parameters between the sham and CPAP groups, but a difference of change in GWW (*p* = 0.045) and GWE (*p* = 0.024) was confirmed between these two groups (Fig. 3).

## Discussion

This randomized, sham-controlled clinical trial demonstrated the association between hypoxemia severity and myocardial work using the LV P-S loop and assessed the benefits of CPAP therapy in patients with severe OSA. The key findings were as follows: (1) in patients with severe OSA, the severity of hypoxemia was more closely associated with myocardial performance than the most commonly used OSA metric, AHI; (2) CPAP treatment for 3 months improved LV myocardial performance by decreasing LV wasted work and increasing work efficacy; (3) GWW increased and GWE decreased during exercise in patients with severe OSA, and CPAP treatment reduced GWW and increased GWE in these patients.

Hypoxemia leads to hypoxia, which causes endothelial dysfunction by causing oxidative stress and inflammation in endothelial cells and mitochondria by altering various intracellular molecules^18^. Endothelial dysfunction is a major cause of cardiovascular disease. OSA is associated with intermittent hypoxia, which increases proangiogenic and pro-inflammatory molecules in cellular model and elevates blood pressure clinically, implying that it increases the LV afterload^19,20^. Thus, intermittent hypoxia in patients with OSA is a major factor that induces endothelial dysfunction and inflammation, ultimately affecting left ventricular diastolic and systolic function.

AHI is the most commonly used index for diagnosis and determining OSA severity (mild: 5–14/h, moderate: 15–29/h, severe: > 30/h)^2^. Although AHI mostly correlates with hypoxemia severity, in some patients whose AHI is mostly attributed to arousal-based hypopneas (without much desaturation), the correlation between the two can be poor. Several studies have reported that hypoxemia severity is more closely related to cardiovascular disease than AHI; in particular, T90 and mean SpO_2_, which are part of the conventional OSA metrics, have been identified as important predictors of sudden cardiac death and mortality in patients with chronic heart failure^21,22^. Similarly, myocardial work was more correlated with hypoxic indices in this study than AHI within the severe OSA category. This suggests that hypoxemia severity may be an important indicator of myocardial dysfunction in patients with severe OSA. Therefore, it is important to evaluate hypoxemia severity, in addition to AHI alone, when examining the relationship between the severity of OSA and the risk of end-organ damage. Subgroup analysis showed a significant difference in the changes in GWW and GWE between the CPAP and sham groups in patients with mean SpO_2_ < 95%. However, in the case of T90 ≥ 10%, GWE increased significantly after CPAP treatment. Although there was no significant difference in any subgroups based on hypoxemia severity, GWW tended to decrease and GWE tended to increase in the CPAP group. Thus, hypoxemia appears to play a major mechanism in the negative effect of OSA on myocardial mechanics, and the severity of the hypoxemia is a predictor of the CPAP effect.

Combined ventricular systolic and arterial stiffening is considered to be an important factor in the pathophysiology of heart failure with preserved ejection fraction^23^. In our previous study, we demonstrated that 3 months of CPAP treatment improved pulse wave velocity, arterial elastance, and ventricular–vascular coupling index^9^. Myocardial work is considered to be the ideal parameter to reflect LV function by mirroring the pressure loading of the LV^13^. In this respect, the improvement of GWE observed in the study is a result of the reduction of LV afterload and activation of the sympathetic nervous system caused by CPAP treatment and the increase in stroke volume and cardiac function^24^. In the subgroup analysis, the effect of GWE and GWW improvement between the CPAP and sham groups was significant in patients with hypertension. This additionally implies that improvement in arterial stiffness is an important factor in the improvement of myocardial work in patients with sleep apnea.

Few studies have evaluated myocardial work during exercise stress echocardiography in healthy populations. In the healthy control individuals in these studies, GWI, GCW, and GWW increased, but there was no change in GWE^25,26^. However, patients with heart failure with preserved ejection fraction showed that the reduced GWE during effort was mainly determined by increased GWW during exercise^27^. In our study, consistent with previous literature, GWI, GCW, and GWW increased during exercise and, as a result, GWE decreased compared to that at baseline. When comparing myocardial work during exercise before and after treatment in each group, there was no significant difference in GWE. However, because GWW was decreased in the CPAP treatment group at 25 W exercise, it was expected that CPAP could improve myocardial function in patients with severe OSA during exercise. However, the underlying mechanism remains unclear. A possible explanation is that OSA is independently associated with clinical and subclinical myocardial ischemia and GWW sensitively reflects myocardial injury. While GWW represents work performed by the LV, it does not contribute to LV ejection, but provides information on myocardial contractile reserve in viable myocardium. Clinically or sub-clinically damaged myocardium has dyssynchronous contractions and post-systolic shortening, which contribute to changes in GWW^28^. Moreover, GWW is related to increased myocardial wall stress against a higher afterload; thus, the damaged myocardium may have been more affected by increased afterload during exercise^29^.

This study had some limitations. First is the innate limitation of the method  used for calculating myocardial work. Myocardial work was calculated assuming that peripheral blood pressure values were equal to central blood pressure and the myocardial strain was equal to volume change. Second, patients with structural heart disease, coronary heart disease, arrhythmia, and uncontrolled BP were excluded. It is not easy to generalize our results across all populations or clinical situations. However, we found that CPAP treatment improved LV mechanics even in patients with subclinical myocardial dysfunction. Third, since the effect of CPAP treatment was compared over a relatively short period of 3 months, the long-term effects are unknown. However, importantly, CPAP therapy improved myocardial performance, even over the short study period. Fourth, the study population was small. Thus, further studies are needed to verify the results using a greater number of patients. However, given the difficulty of conducting a randomized sham-controlled trial of CPAP on a large number of patients, the results of this study are still valuable. Finally, no comparison was made with a healthy population. Therefore, it is difficult to generalize the value of myocardial work in this study to the characteristics of patients with OSA. Nevertheless, this study is meaningful as it confirmed the correlation between the severity of hypoxemia and LV performance using myocardial work in patients with severe OSA.

In conclusion, indices of hypoxic burden are more closely associated with myocardial performance than AHI in patients with severe OSA. 3-months CPAP treatment improved LV myocardial performance by decreasing LV wasted work and increasing work efficacy compared to the sham treatment.

## Methods

### Study population and design

This study was conducted as a sub-study of the previous trial: The Continuous Positive Airway Pressure Therapy on Left Ventricular Diastolic Function in Patients with Severe Obstructive Sleep Apnea trial was designed to investigate the effects of 3 months of CPAP on LV diastolic function. It was a prospective, single-center, randomized, blinded, sham-controlled trial of parallel groups. The design and primary outcomes of the trial have been previously reported^9^. Briefly, a total of 60 patients with severe OSA (AHI > 30/h) by screening polysomnography (PSG) were enrolled^2^. Exclusion criteria included LV ejection fraction < 50%, uncontrolled blood pressure (BP) (≥ 180/110 mmHg), prior history of coronary artery disease, significant valvular dysfunction (of a moderate or greater degree), arrhythmia, an estimated glomerular filtration rate of less than 60 mL/min/1.73 m^2^ of body surface area, and treatment with anxiolytics or sedatives. The final 56 patients were randomly divided into a sham treatment group (n = 28) and a CPAP treatment group (n = 28) through 1:1 randomization (Supplementary Fig. S2). All patients were masked to the treatment.  Over the 3-months treatment period, two patients in each group discontinued interventions; thus, 26 patients in each group were included in the analysis. The data on study populations had been used to assess diastolic function and speckle tracking echocardiography in a previous study^9,10^ and were re-analyzed to calculate myocardial work parameters in the current study. The original trial is registered with www.ClinicalTrials.gov (NCT01854398). This study was approved by the Institutional Review Board of the Yonsei University Health System (approval number: 1-2013-0021), and the investigation conforms with the principles outlined in the Declaration of Helsinki. All participants provided informed consent.

### Measures of obstructive sleep apnea severity

The standard channels recommended by the American Academy of Sleep Medicine (AASM) were used to perform overnight PSG, and data were processed using the Embletta X100 system (Natus Embla Systems, Oakville, ON, Canada). AHI was defined as the number of apnea and hypopnea events divided by total sleep time, expressed as events per hour. Apnea was defined as a reduction in airflow greater than 90% of the pre‐event baseline and occurring for ≥ 10 s measured using a thermocouple signal. Hypopnea events were defined as a reduction in airflow > 30% of the pre‐event baseline and occurring for ≥ 10 s in association with at least a 3% oxygen desaturation or arousal. Hypopnea was considered obstructive if any of the following were present: snoring during the event, an increase in the flattening of the nasal pressure flow, or paradoxical breathing. Obstructive AHI ≥ 15 events/h was considered clinically significant for OSA. Central apneas were defined as apneas occurring without associated respiratory effort lasting ≥ 10 s.

Severe OSA was defined as an AHI > 30/h, and very severe OSA was defined as an AHI > 50/h in this study^30,31^. Measures of PSG-derived hypoxemia metrics, including mean SpO_2_ during sleep, T90, and ODI, were used to further classify patients with severe OSA into those with and without severe hypoxemia.

### Transthoracic echocardiography and exercise echocardiography

All patients underwent transthoracic echocardiography at baseline and after 3 months in both the CPAP and sham treatment groups using a 2.5-MHz transducer ultrasound machine (Vivid 7 or Vivid E9 system, GE Healthcare). Conventional 2D, M-mode, and Doppler images were obtained and measured according to the current recommendations^32^. 2D speckle tracking echocardiography was performed using customized software (EchoPAC PC, version 113; GE Medical Systems). We manually traced the endocardial border of the LV in the acquired apical four-chamber, apical three-chamber, and apical two-chamber views to obtain the LVGLS. The LVGLS was calculated by averaging the peak strain value of the three apical views. All data were measured by two experienced cardiologists who were blinded to the data analysis.

Exercise stress echocardiography was performed for all patients at baseline and the 3-month follow-up. Symptom-limited supine bicycle exercise stress echocardiography was performed according to current guidelines using a cycle ergometer^33^. The participants started pedaling at a workload of 25 W, which was increased by 25 W every 3 min up to peak exercise and stopped if the patient complained of symptoms. Three standard apical views were acquired at each exercise stage. Echocardiographic images at 25 W and 50 W were used for analysis, as the measurement results were unreliable due to the poor quality of speckle tracking in tachycardia conditions (almost ≥ 75 W images).

### Myocardial work evaluation

Myocardial work was calculated as a combination of LV strain and the estimated LV pressure curve. In the software, the LV strain provided the P–S loop, which was calculated using 2D speckle tracking echocardiography, and peripheral systolic blood pressure was measured non-invasively under the assumption that the peak LV pressure was equal to the peak systolic arterial pressure. The isovolumic and ejection phases were determined through the opening and closing times of the mitral and aortic valves^16^. The calculated myocardial work parameters were as follows:

1. Global work index (GWI): total work of LV, that is, the area under the curve of the P–S loop.
2. Global constructive work (GCW): work of LV that contributes to LV ejection during systole, that is, myocardial shortening during systole (positive segmental work) and lengthening during isovolumic relaxation (negative segmental work).
3. Global wasted work (GWW): work of LV that does not contribute to LV ejection, specifically, myocardial lengthening during systole (negative segmental work) and shortening during isovolumic relaxation (positive segmental work).
4. Global work efficiency (GWE): the ratio of constructive work to total work (GCW/[GCW + GWW]).

The BP of all patients was measured in the supine position after a 5-min rest just before echocardiography. Supplementary Fig. S3 illustrates the measurement of the myocardial work parameters using the software.

### Statistical analysis

As described previously, the sample size was calculated using an a-value of 0.05, a beta value of 0.1, and a power of 90% based on previous research. Assuming a dropout rate of 20%, the sample size was calculated as 30 patients per group, resulting in a target sample size of 60 patients^7^. Continuous variables are presented as mean ± standard deviation, and categorical variables are presented as frequencies (%). Between-group comparisons were analyzed using a two-sample t-test. Before and after treatment comparisons were performed using paired t-tests. Pearson’s correlation analysis and multivariable regression analysis were used to analyze the correlation between OSA severity and LV performance. Subgroup analysis was performed by dividing patients based on the presence or absence of hypertension and by the median value of OSA severity indices, such as AHI, mean SpO_2_, T90, and ODI. Statistical significance was set at a two-sided p-value < 0.05. Statistical analyses were conducted using SPSS (version 22.0; IBM, Armonk, NY, USA) and R statistical software (version 4.0.2; R Foundation for Statistical Computing, Vienna, Austria).

## Supplementary Information

Supplementary Information.

## Data Availability

Data of this study are available from the corresponding author on request due to privacy/ethical restrictions.
