# Supplementary material for: Effects of continuous positive airway pressure therapy on left ventricular performance in patients with severe obstructive sleep apnea
Source: Sci Rep. 2023 Apr 1;13:5335. doi: 10.1038/s41598-023-32274-4 (PMC10067829; doi:10.1038/s41598-023-32274-4)

Supplementary Information

**Effects of continuous positive airway pressure therapy on left ventricular performance in patients with severe obstructive sleep apnea**

Se-Eun Kim,^1‡^ Jiwon Seo,^1‡^ Younghoon Kwon,^2^ Iksung Cho,^1^ Chi Young Shim,^1^ Jong-Won Ha,^1^ Geu-Ru Hong^1*^

^1^Division of Cardiology, Severance Cardiovascular Hospital, Yonsei University College of Medicine, Seoul, Korea

^2^Division of Cardiology, University of Washington Seattle, WA 98104

‡These authors contributed equally to this work.

***Corresponding Author:**

Geu-Ru Hong, M.D., Ph.D.

Division of Cardiology, Severance Cardiovascular Hospital, Yonsei University College of Medicine, 50-1 Yonsei-ro, Seodaemun-gu, Seoul, Republic of Korea 03722

Phone: +82-2-2228-8453, Fax: +82-2-2227-7742, E-mail: GRHONG@yuhs.ac

**Supplementary Table 1. Changes of GCW, GWW, and GWE in the subgroups.**

|  | **Sham** | **CPAP** | **P-value** |
| --- | --- | --- | --- |
| **Overall** |  |  |  |
| **Subjects, n** | 26 | 26 |  |
| **Change of GCW, mmHg%** | -48.3 ± 370.8 | -48.9 ± 347.8 | 0.996 |
| **Change of GWW, mmHg%** | 17.1 ± 43.2 | -18.9 ± 33.8 | 0.003 |
| **Change of GWE, %** | -1.2 ± 2.8 | 1.7 ± 3.4 | 0.003 |
| **Without HTN** |  |  |  |
| **Subjects, n** | 11 | 9 |  |
| **Change of GCW, mmHg%** | -60.9 ± 211.5 | -264.6 ± 274.8 | 0.094 |
| **Change of GWW, mmHg%** | 29.0 ± 61.6 | -15.1 ± 36.4 | 0.093 |
| **Change of GWE, %** | -1.4 ± 3.5 | 0.6 ± 2.4 | 0.185 |
| **With HTN** |  |  |  |
| **Subjects, n** | 15 | 17 |  |
| **Change of GCW, mmHg%** | -38.7 ± 467.2 | 66.2 ± 333.9 | 0.496 |
| **Change of GWW, mmHg%** | 7.9 ± 19.3 | -20.9 ± 33.4 | 0.011 |
| **Change of GWE, %** | -1.1 ± 2.2 | 2.3 ± 3.8 | 0.010 |
| **T90 ≤10%** |  |  |  |
| **Subjects, n** | 18 | 17 |  |
| **Change of GCW, mmHg%** | -11.1 ± 405.5 | -119.2 ± 286.9 | 0.391 |
| **Change of GWW, mmHg%** | 9.9 ± 20.2 | -14.9 ± 28.6 | 0.008 |
| **Change of GWE, %** | -0.5 ± 2.5 | 1.0 ± 2.2 | 0.081 |
| **T90 >10%** |  |  |  |
| **Subjects, n** | 8 | 9 |  |
| **Change of GCW, mmHg%** | -133.4 ± 284.4 | 112.0 ± 440.8 | 0.239 |
| **Change of GWW, mmHg%** | 33.6 ± 73.3 | -28.1 ± 44.7 | 0.082 |
| **Change of GWE, %** | -2.9 ± 2.9 | 3.3 ± 5.2 | 0.017 |
| **Mean SpO_2_ ≤95%** |  |  |  |
| **Subjects, n** | 16 | 19 |  |
| **Change of GCW, mmHg%** | -6.9 ± 374.5 | -50.1 ± 356.9 | 0.746 |
| **Change of GWW, mmHg%** | 23.7 ± 51.6 | -17.7 ± 31.4 | 0.010 |
| **Change of GWE, %** | -1.6 ± 3.1 | 1.5 ± 3.6 | 0.018 |
| **Mean SpO_2_ >95%** |  |  |  |
| **Subjects, n** | 10 | 7 |  |
| **Change of GCW, mmHg%** | -112.8 ± 377.4 | -45.5 ± 352.9 | 0.734 |
| **Change of GWW, mmHg%** | 6.8 ± 24.9 | -22.3 ± 43.1 | 0.119 |
| **Change of GWE, %** | -0.7 ± 2.3 | 2.3 ± 3.1 | 0.051 |
| **AHI (≤50)** |  |  |  |
| **Subjects, n** | 12 | 8 |  |
| **Change of GCW, mmHg%** | -7.2 ± 436.9 | -222.6 ± 229.9 | 0.254 |
| **Change of GWW, mmHg%** | 10.0 ± 18.9 | -7.1 ± 28.7 | 0.157 |
| **Change of GWE, %** | -0.6 ± 2.4 | 0.4 ± 2.2 | 0.381 |
| **AHI (>50)** |  |  |  |
| **Subjects, n** | 14 | 18 |  |
| **Change of GCW, mmHg%** | -80.0 ± 326.2 | 27.1 ± 368.8 | 0.420 |
| **Change of GWW, mmHg%** | 22.5 ± 55.6 | -24.1 ± 35.4 | 0.011 |
| **Change of GWE, %** | -1.7 ± 3.0 | 2.2 ± 3.8 | 0.005 |
| **ODI (≤39)** |  |  |  |
| **Subjects, n** | 15 | 11 |  |
| **Change of GCW, mmHg%** | -13.2 ± 438.3 | -126.7 ± 300.5 | 0.491 |
| **Change of GWW, mmHg%** | 10.5 ± 20.5 | -14.1 ± 35.6 | 0.049 |
| **Change of GWE, %** | -0.4 ± 2.5 | 1.4 ± 2.8 | 0.127 |
| **ODI (>39)** |  |  |  |
| **Subjects, n** | 9 | 13 |  |
| **Change of GCW, mmHg%** | -150.2 ± 223.2 | 57.0 ± 398.4 | 0.182 |
| **Change of GWW, mmHg%** | 24.2 ± 65.8 | -24.8 ± 35.7 | 0.047 |
| **Change of GWE, %** | -2.2 ± 2.9 | 2.3 ± 4.2 | 0.014 |

Values are n (%) or mean ± standard deviation.

AHI, apnea/hypopnea index; AI (HR 75), augmented index corrected for heart rate of 75 bpm; GCW, global constructive work; GWE, global work efficiency; GWW, global wasted work; HTN, hypertension; Mean SpO_2_, average O_2_ saturation during sleep; ODI, oxygen desaturation index; T90, percentage of sleep time below 90% oxygen saturation.

**Supplementary Table 2. Myocardial work during exercise**

|  | **Baseline** | **25W** | **P-value*** | **50W** | **P-value**† |
| --- | --- | --- | --- | --- | --- |
| **GWI, mmHg%** | 1582.3 ± 290.5 | 1872.9 ± 344.2 | <0.001 | 2028.6 ± 352.4 | <0.001 |
| **GCW, mmHg%** | 1794.4 ± 307.4 | 2433.8 ± 432.1 | <0.001 | 2630.3 ± 505.0 | <0.001 |
| **GWW, mmHg%** | 75.2 ± 44.3 | 184.4 ± 125.4 | <0.001 | 171.7 ± 105.5 | <0.001 |
| **GWE, %** | 94.6 ± 3.9 | 92.1 ± 4.3 | 0.004 | 92.9 ± 3.7 | 0.033 |

***** Paired t-test, compared with the value at baseline and at 25W

† Paired t-test, compared with the value at baseline and at 50W

GCW, global constructive work; GWE, global work efficiency; GWI, global work index; GWW, global wasted work.

**Supplementary Table 3. Change of myocardial work during exercise**

|  | **Baseline** | | | **Follow-up** | | |
| --- | --- | --- | --- | --- | --- | --- |
|  | **Sham** | **CPAP** | **P-value** | **Sham** | **CPAP** | **P-value** |
| **25W** | | | | | | |
| **LVGLS, %** | -17.2 ± 2.2 | -17.8 ± 2.6 | 0.401 | -17.6 ± 1.5 | -17.7 ± 2.1 | 0.832 |
| **GWI, mmHg%** | 1855.6 ± 319.3 | 1891.1 ± 375.2 | 0.734 | 1749.3 ± 258.1**^†^** | 1704.3 ± 302.2 | 0.593 |
| **GCW, mmHg%** | 2397.0 ± 354.1 | 2472.1 ± 506.7 | 0.566 | 2252.9 ± 304.2 | 2186.9 ± 341.4 | 0.496 |
| **GWW, mmHg%** | 179.0 ± 129.0 | 190.1 ± 124.4 | 0.77 | 193.8 ± 99.5 | 140.7 ± 58.2**^†^** | 0.035 |
| **GWE, %** | 92.3 ± 4.8 | 91.9 ± 3.7 | 0.71 | 91.6 ± 3.3 | 93.0 ± 2.1 | 0.101 |
| **50W** | | | | | | |
| **LVGLS, %** | -18.3 ± 2.1 | -18.2 ± 2.8 | 0.914 | -17.7 ± 2.1 | -18.0 ± 2.3 | 0.603 |
| **GWI, mmHg%** | 2017.6 ± 348.8 | 2040.0 ± 364.3 | 0.838 | 1775.9 ± 352.0**^†^** | 1851.3 ± 322.6 | 0.464 |
| **GCW, mmHg%** | 2612.5 ± 474.9 | 2648.9 ± 545.9 | 0.816 | 2437.7 ± 379.6 | 2409.8 ± 485.9 | 0.832 |
| **GWW, mmHg%** | 174.1 ± 122.2 | 169.1 ± 87.6 | 0.878 | 212.8 ± 113.1 | 156.0 ± 96.8 | 0.082 |
| **GWE, %** | 92.8 ± 4.2 | 93.0 ± 3.3 | 0.877 | 91.2 ± 3.9 | 93.1 ± 3.2 | 0.086 |

†Paired t-test, P<0.05, compared with the corresponding baseline group

GCW, global constructive work; GWE, global work efficiency; GWI, global work index; GWW, global wasted work; LVGLS, left ventricular global longitudinal strain.

**Supplementary Figure 1.** Representative figure of the measurement of myocardial work parameters using the software.


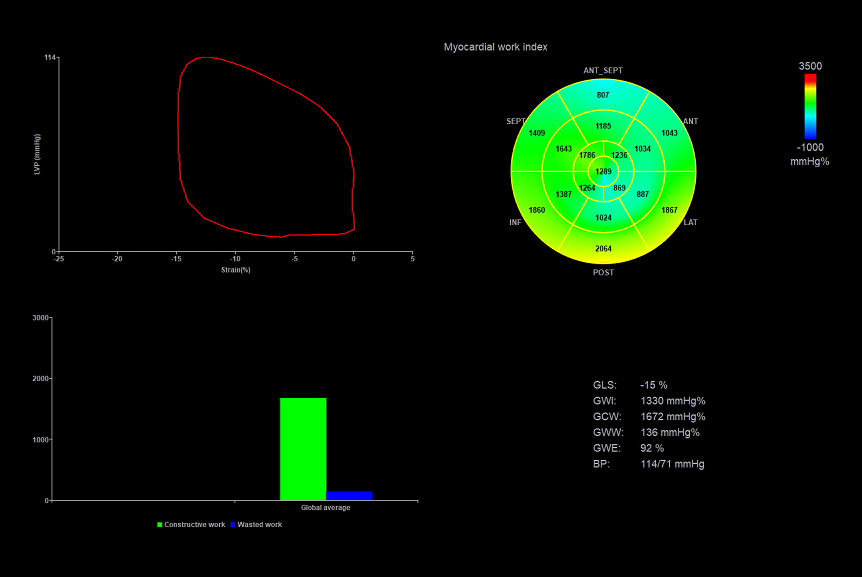


**Supplementary Figure 2.** Changes in GWW and GWE in the two study groups. CPAP, continuous positive airway pressure; GWE, global work efficiency; and GWW, global wasted work.


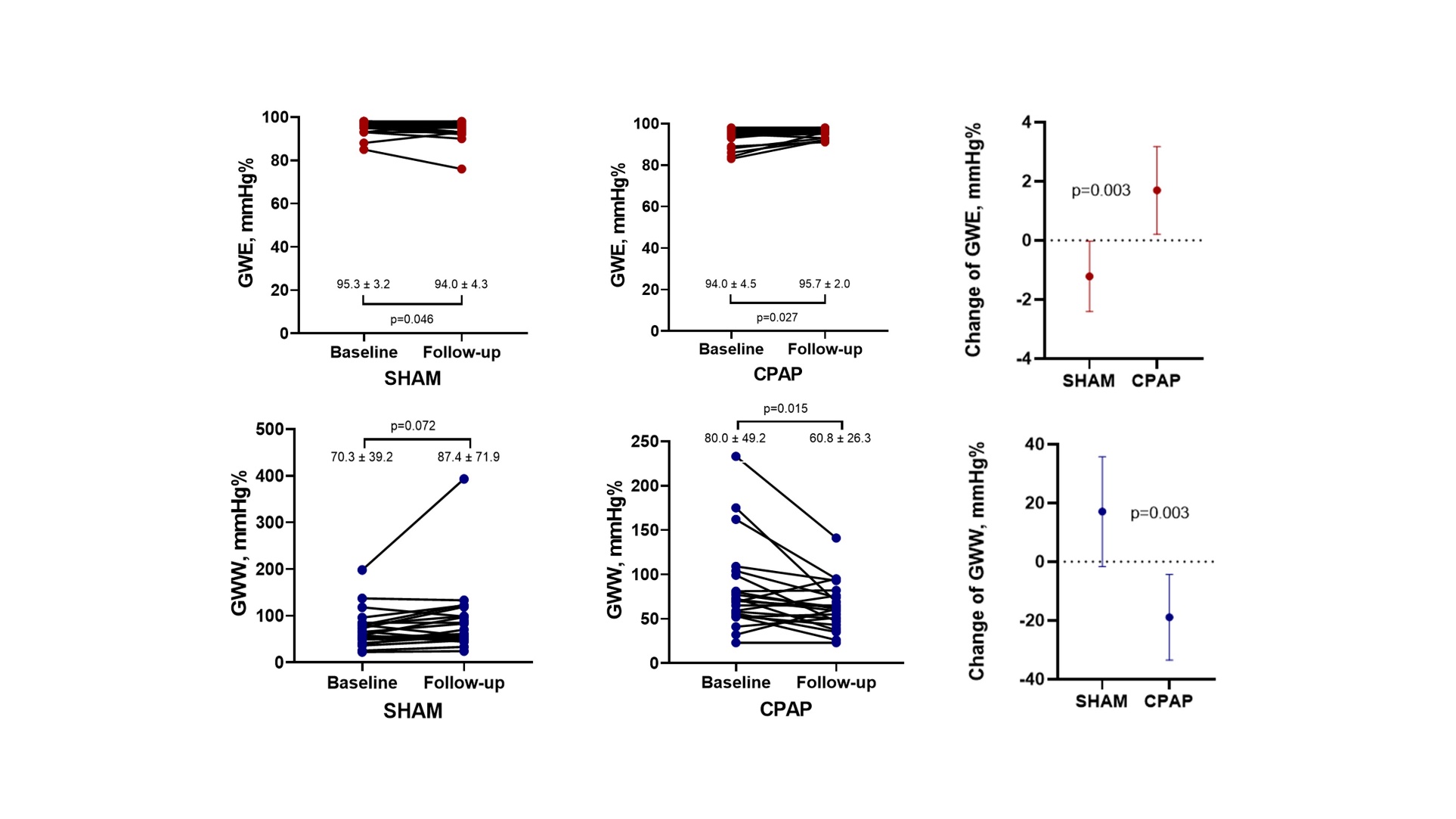


**Supplementary Figure 3.** Flow chart of the study process.


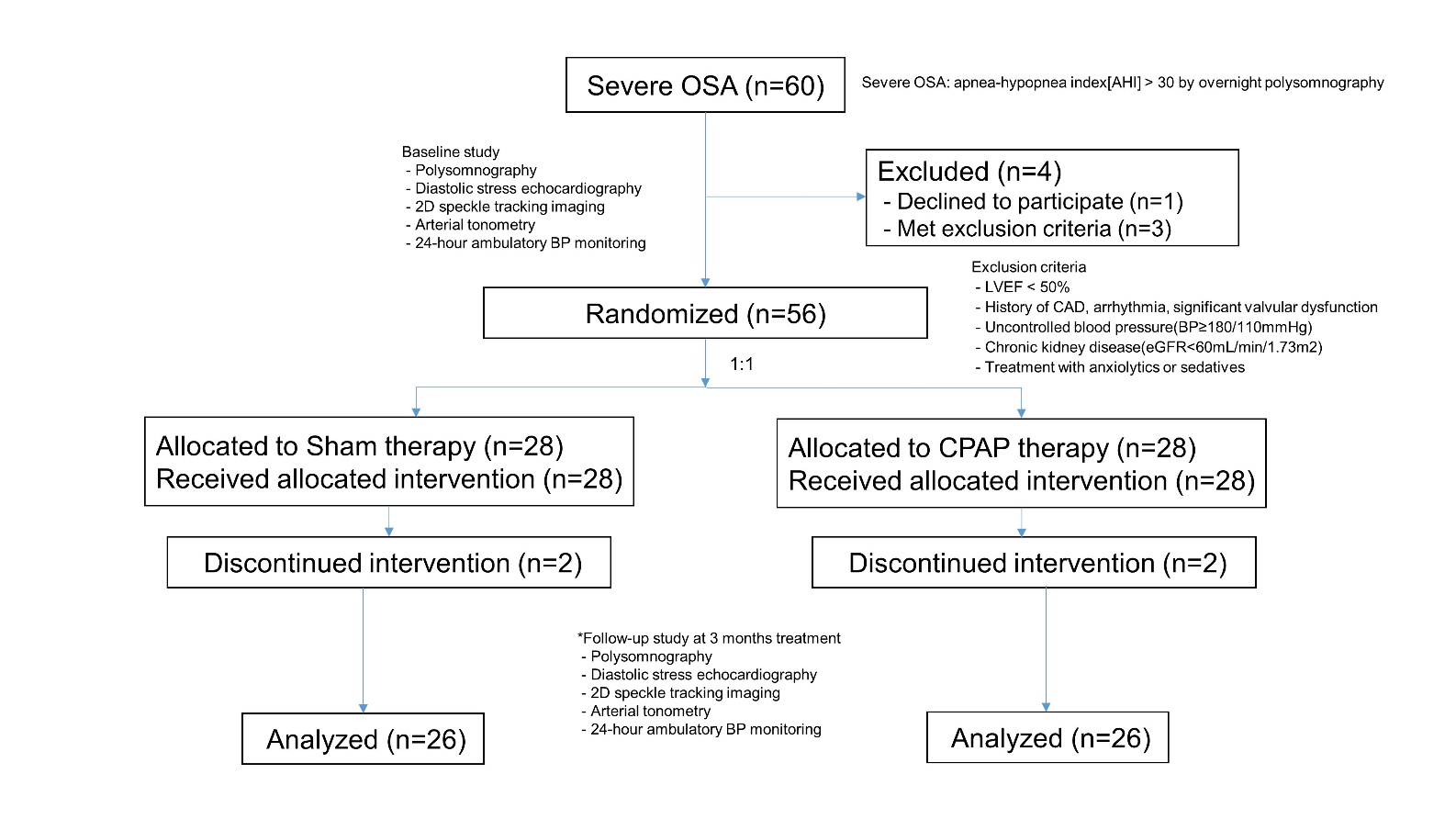

Supplement: Supplementary file 1 — Supplementary Information. [file 41598_2023_32274_MOESM1_ESM.docx]
